# Identification of Subtype-Specific Three-Gene Signature for Prognostic Prediction in Diffuse Type Gastric Cancer

**DOI:** 10.3389/fonc.2019.01243

**Published:** 2019-11-12

**Authors:** Bowen Bao, Chunlei Zheng, Bowen Yang, Yue Jin, Kezuo Hou, Zhi Li, Xueying Zheng, Shitong Yu, Xiaojie Zhang, Yibo Fan, Xiujuan Qu, Yunpeng Liu, Xiaofang Che

**Affiliations:** ^1^Department of Medical Oncology, The First Hospital of China Medical University, Shenyang, China; ^2^Key Laboratory of Anticancer Drugs and Biotherapy of Liaoning Province, The First Hospital of China Medical University, Shenyang, China; ^3^Liaoning Province Clinical Research Center for Cancer, Shenyang, China

**Keywords:** gastric cancer, diffuse type, signature, nomogram, peritoneal metastasis

## Abstract

Gastric cancer (GC), with high heterogeneity, can be mainly classified into intestinal type and diffuse type according to the Lauren classification system. Although a number of differences were reported between these two types, no study on the Lauren subtype-specific multi-gene signature for evaluation of GC prognosis has been conducted, and the molecular mechanism underlying its poor prognosis has still remained elusive. Therefore, this study aimed to explore subtype-specific multi-gene signature for prognostic prediction in different subtypes of Lauren classification. With combination of the least absolute shrinkage and selection operator (LASSO) algorithm and the Akaike information criterion (AIC), the 3-gene subtype-specific prognostic signature was successfully established in diffuse type GC using GSE62254 dataset. Following the calculation of risk score (RS) based on 3-gene signature, the nomogram models were established to predict 1-, 3-, and 5-year overall survival in diffuse type GC. Moreover, the prognostic predictive nomogram model of diffuse type GC was also proved to be effective for validation of GSE1549 dataset and by a Gene Expression Omnibus (GEO)-based meta-analysis. In the analysis of the correlation between RS and clinical-pathological characteristics, RS and two genes of the 3-gene signature (EMCN and COL4A5) were found to be positively correlated with peritoneal metastasis. Furthermore, EMCN and COL4A5, rather than CCL11, were proved to be able to enhance the adhesion ability of MKN45 and NUGC4 cells to peritoneal mesothelial cell line HMR-SV5. Eventually, it was proved that COL4A5 promoted peritoneal metastasis by activating Wnt signaling pathway, whereas the upregulation of integrin family genes mediated by FAK-AKT/ERK/STAT3 signaling pathway activation is involved in peritoneal metastasis promotion function of EMCN. Taken together, our study identified the subtype-specific 3-gene signature in diffuse type GC, which could effectively predict the patients' OS and might explain the molecular mechanisms in presence of its poor prognosis.

## Introduction

Gastric cancer (GC), the third leading cause of cancer deaths worldwide, seriously threatens human health (1). The incidence of GC is the fifth highest among different types of cancer, and that is more frequent in Eastern Asia, especially in China (2). Although surgical therapy may lead to 5-year survival rates of 80–100% for patients who are in early stage, the majority of patients are in advanced stages at their initial diagnosis, thereby losing the opportunity for surgery. Despite rapid development of treatments for GC, an insignificant progress has been achieved in terms of effective therapeutics for advanced GC due its high heterogeneity, in which the median overall survival (OS) is still shorter than 1 year (3).

GC can be classified into different subtypes according to different classification systems, such as the Bormann, the Lauren, and the World Health Organization (WHO) classification systems, indicating the high heterogeneity of GC (4–10). Among these classification systems, Lauren classification, mainly including intestinal- and diffuse type, is extensively used in clinical practice. The greatest advantage of Lauren classification is that it is easy to perceive the histology and biology of GC. In histology, intestinal type GC cells exhibit a tubular or glandular differentiation with a more intensive arrangement and a tighter adhesion junction, whereas diffuse type GC cells are typically scattered and have poor adhesion ability, thereby causing lack of gland formation and easy to dissemination. The incidence rate and prognosis of these two types is also different, in which intestinal type is the most prevalent type with a higher 5-year survival rate and a further frequent incidence in men and older patients, while diffuse type is lower in the incidence and shorter in duration of OS. However, although those apparent differences existed between the two types, Lauren classification system is still rarely utilized in the clinical practice due to lack of significant difference in their prediction and treatment capabilities. Therefore, it is essential to explore the subtype-specific molecular mechanisms in intestinal- and diffuse types of GC.

It has been reported that the number of genes is different in the expression and function between the two types. HER2, a classical gene targeted by Trastuzumab, was identified with a higher positive rate in intestinal type GC (11). FGFR2 was found to be associated with poor prognosis of diffuse type GC cells (12). Remarkable expression of E-cadherin and TP53 was also related to the diffuse- and intestinal type GC, respectively (13). In addition, the incidence of microsatellite instability (MSI) in intestinal type was reported to be higher than that in diffuse type (14). However, development of GC depends on the regulation of multiple signaling pathways, and a single gene is difficult to illustrate the difference between the two types. Therefore, it is of great importance to identify subtype-specific multi-gene signatures to predict prognosis and perceive the molecular difference in intestinal and diffuse types of GC.

In the present study, GSE62254 dataset extracted from the Gene Expression Omnibus (GEO) database was used to identify 3- and 5-gene prognostic signatures, and specific prognostic predictive nomogram models were established in diffuse- and intestinal type GC, respectively. Furthermore, the prognostic value of 3-gene signature in diffuse type, rather than 5-gene signature in intestinal type, was also validated in GSE1549 dataset by a GEO-based meta-analysis. Moreover, the 3-gene signature was found to be associated with peritoneal metastasis. These outcomes revealed molecular characteristics and biological mechanisms under poor prognosis, and may provide a reliable reference for the treatment of diffuse type GC.

## Materials and Methods

### Data Collection and Patient Information

Microarray datasets GSE62254 was downloaded from the Gene Expression Omnibus (GEO) database (http://www.ncbi.nlm.nih.gov/geo/), which was used as a training set for prognostic prediction of the multi-gene signature. The samples with Lauren subtypes were filtered by the criteria that owned integral clinical parameters and survival data (15). The detailed clinical data of these datasets are shown in supplementary materials (Table S1). The RMA algorithm was performed to normalize and transform all the raw data from GEO to expression values in the R environment (v3.5.3) (16).

### Differentially Expressed Gene Analysis and Candidate Genes Identification

Differentially expressed genes (DEGs) between diffuse and intestinal subtype samples were screened with the thresholds of Q value (adjusted *P*-value between two groups) <0.05 and |Log fold change (FC)| > 0.585 using the “limma” package in R (17). The Log FC of DEG genes more than 0.585 was identified as the diffuse subtype-specific genes, whereas that <-0.585 was intestinal subtype-specific genes. To identify the gene with prognostic value, the univariate Cox regression analysis was applied using “survival” package. The HRs and their corresponding *P*-value of all genes in the GSE62254 datasets were obtained under the univariate Cox regression. The genes with *P* < 0.05 were defined to be related with the over survival. Then, the overlapping genes between the two subtype DEG genes and OS-related genes were picked up as candidate genes, and venn diagram was carried out using Venny 2.1.0.

### The Construction of Multi-Gene Signature Risk Score Model Based on LASSO Algorithm and AIC

The glmnet package in R was utilized to perform the COX regression analysis with LASSO algorithm (Least Absolute Shrinkage and Selection Operator) (18). The robust markers were selected from candidate genes in two subtypes by LASSO algorithm, in which the datasets were subsampled and the tuning parameters were determined according to the expected generalization error estimated from 10-fold cross-validation. Then, a multivariate Cox regression analysis with stepwise method based on the AIC (Akaike information criterion) calculation was conducted to screen the independent prognostic factors in those robust markers. The risk score (RS) was established for each patient by calculating the expression values of the selected genes weighted by their corresponding coefficients in the multivariate Cox regression analysis.

### The Establishment of Nomogram Models

The samples were divided into low-RS and high-RS groups according to the median RS. Using R with package “survival,” Kaplan–Meier was performed to show the relationships between RS and the survival time, and the Log-rank test was utilized to analyze the differences between groups. After the multivariate Cox regression analysis for the selection of independent prognostic factor, RS and others clinical pathological characteristics were used to generate the nomogram and calibration plots by “rms” package in R. In this model, each factor was assigned a weight score based on the results of the multivariate Cox regression analyses. Calibration was used to assess the performance of the nomogram. Receiver operating characteristic (ROC) analysis was also performed to estimate the accuracy of the nomogram for survival prediction using the “survival ROC” package of R. In addition, C-index was calculated with “survival” package.

### External Validation of Multi-Gene Signature RS Mod el by GEO Meta-Analysis

Kaplan–Meier with the log-rank test was applied to show the survival difference between high-RS and low-RS groups in datasets GSE15459 (19). Furthermore, the microarray datasets relevant to Lauren subtype in gastric cancer tissues published up to May 1st, 2019 were searched in GEO database, and only the datasets with integral Lauren subtype information and survival data were preserved (20). The RS and its corresponding OR and 95% CIs in these datasets was analyzed by the package “meta” in R.

### Clinical Relation Analysis and Biological Function Prediction

Chi-square was applied with “stats” in R between RS or every single gene of the multi-genes and other clinical pathological characteristics. The significance was defined as *P* < 0.05. To explore the biological function of prognosis signature, GSEA was performed using a Java GSEA desktop application that was downloaded from http://www.broad.mit.edu/gsea/ (21). The GSE62254 dataset was analyzed with the GMT file (c2.KEGG.v6.2) gene set to obtain biological processes enriched by biomarkers in prognosis signature. A total of four files including expression datasets, gene sets, phenotype labels and chip platforms were loaded for running GSEA according to the manufacturer's specifications. False discovery rate (FDR) < 0.25 were identified to be significantly enriched and the significantly enriched KEGG pathways were visualized using R package “ggplot2.”

### Cell Line and Cell Culture

The diffuse type GC cell lines MKN45 (3111C0001CCC000229) was obtained from the National Infrastructure of Cell Line Resource (Beijing, China), and NUGC4 (JCRB0834) was from JCRB cell bank (Osaka, Japan). The human peritoneal mesothelial cell line HMR-SV5 was gifted from Prof. Huimian Xu (Department of Surgical Oncology and General Surgery, The First Hospital of China Medical University). All the cells were cultured in RPMI-1640 medium containing 10% heat-inactivated FBS at 37°C under 5% CO_2_ and saturated humidity.

### Realtime PCR

The isolation and reverse transcription of RNA was performed as previously described (22). Comparative cycle threshold (Ct) method was used to calculate relative expression of COL4A5 and CCL11, and the expression of 18S was used as the internal control. The PCR primers used were as follows:

COL4A5 forward: TGGACAGGATGGATTGCCAG;
COL4A5 reverse: GGGGACCTCTTTCACCCTTAAAA;
CCL11 forward: TCCCTGGAATCTCCCACACT;
CCL11 reverse: CTGAAGGTGTGAGCTTTGGC;
18S forward: 5′ -CCCGGGGAGGTAGTGACGAAAAAT-3′;
18S reverse: 5′ -CGCCCGCCCGCTCCCAAGAT-3′.

### RNA Interference

The specific siRNAs of COL4A5 and CCL11 and negative control siRNA (NC) were designed and synthesized by ViewSolid Biotech (Beijing, China). siRNA sequences were as follows: siCOL4A5: CAAUAAUGUUUGCAACUUUtt; siCCL11: GCAUGGGUUUUAUUAUAUAtt; NC siRNA: AATTCTCCGAACGTGTCACGT. siRNAs were transfected into cells using Lipofectamine 2000 (Invitrogen) according to the manufacturer's protocol. Cells were harvested 48 h after transfection.

### Adhesion Assay

MKN45 and NUGC4 cells were pre-labeled with 2 μg/ml of DID dye (Invitrogen, Carlsbad, CA, USA) for 1 h, and placed onto the monolayer of HMV-SV5 cells for another 6 h at 37°C. Then, after removing the non-adherent MKN45 and NUGC4 cells with 3-times PBS washing, DID-labeling cells were observed under a fluorescence microscope (Olympus, Tokyo, Japan). Three representative fields were randomly counted and analyzed statistically.

## Results

### Identification of Subtype-Specific Multi-Gene Signatures in Diffuse and Intestinal Type GC

**Figure 1:**
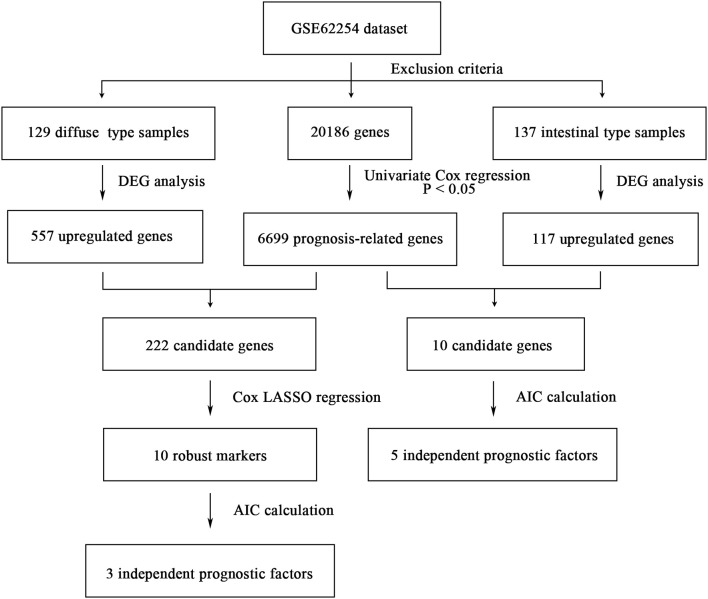
The flowchart of identifying procedure for the multi-gene signatures in intestinal and diffuse type GC.

**Figure 2:**
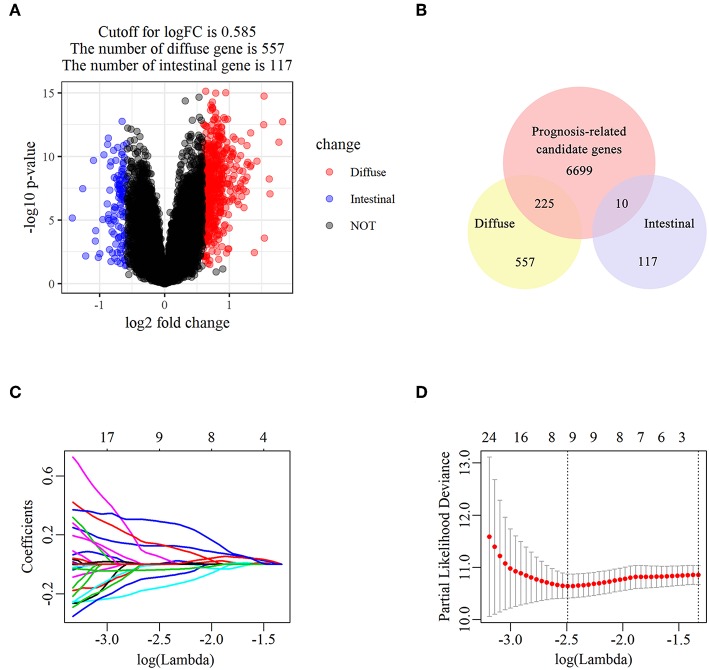
The identification of diffuse type GC-specific multi-gene signature. DEGs between intestinal and diffuse type GC were showed in the volcano plot, in which the red plots represent the genes highly related with diffuse subtype whereas the blue ones represent the genes upregulated in intestinal subtype, while the cutoff for logFC was 0.585 **(A)**. The venn diagram showed that the candidate genes was screened through the intersection of prognosis-related genes with upregulated genes in intestinal and diffuse type GC, respectively **(B)**. The trajectory of each prognosis-related candidate gene's coefficient in diffuse type GC was observed in the LASSO coefficient profiles with the changing of the lambda in LASSO algorithm **(C)**. After the 10-fold cross-validation, a confidence interval was got for partial likelihood deviance as the lambda changed. The dotted line indicated the best gene capacities **(D)**.

The flowchart of screening process for independent prognostic gene markers between diffuse- and intestinal- types of GC is presented in Figure 1. The details were described in the following. Using GSE62254 dataset, 266 samples with definite Lauren subtypes were filtered by the criteria that contained clinical parameters and survival data, including 129 diffuse type GC samples and 137 intestinal type GC samples. Under the criteria that *P* < 0.05 and |LogFC| ≥ 0.585, a total of 674 differentially expressed genes (DEGs), including 557 genes in diffuse type and 117 genes in intestinal type, were screened (Figure 2A). Then, 225 prognosis-related candidate genes (*P* < 0.05) were picked out from DEGs in diffuse type (Figure 2B and Table S2), and 10 candidate genes in intestinal type (Figure 2B and Table S3) by univariate Cox regression analysis. To reduce the high dimension caused by exceeded prognosis-related candidate genes, Cox regression analysis combined with least absolute shrinkage and selection operator (LASSO) algorithm was further conducted for diffuse type GC, and 10 robust markers with non-zero coefficient were identified (Figures 2C,D). Furthermore, followed by choosing the smallest Akaike information criterion (AIC) via the stepwise method (Table S4), the optimal prognostic signatures (“CCL11,” “COL4A5,” and “EMCN”) in diffuse type were determined and nominated as “3-gene signature” (Table 1). On the other hand, the AIC calculation of 10 candidate genes in intestinal type was carried out, and five independent prognostic factors (5-gene signature) were detected in intestinal type (Tables S5, S6). These data indicated that the diffuse- and intestinal type GC, as key factors, were notably different, and multi-gene signatures of diffuse- and intestinal type GC might influence the prognosis of these two types, respectively.

**Table 1:** The univariate and multivariate Cox regression analysis between 10 robust markers and OS in diffuse type GC.

	**Variate COX**				**Multivariate COX**			
	**Coef**	**HR**	***P***		**Coef**	**HR**	***P***	
CCL11	−0.1378	0.8712	0.0403	*	−0.2733	0.7609	0.0015	**
RORA	0.5163	1.6759	0.0004	***				
COL4A5	0.3200	1.3771	0.0002	***	0.1769	1.1936	0.0445	*
A2M	0.5455	1.7255	0.0009	***				
TENT5C	−0.2647	0.7675	0.0232	*				
TLR8	−0.2285	0.7957	0.0186	*				
NR4A3	0.3354	1.3985	0.0014	**				
TUSC3	0.3335	1.3959	0.0004	***				
ACKR4	−0.1860	0.8303	0.0138	*				
EMCN	0.5177	1.6781	0.0002	***	0.4744	1.6071	0.0063	**

^***^P < 0.001; ^**^P < 0.01; ^*^P <0.05.

### Establishment and Evaluation of the Prognostic Predictive Nomogram Model in Diffuse- and Intestinal Type GC

**Figure 3:**
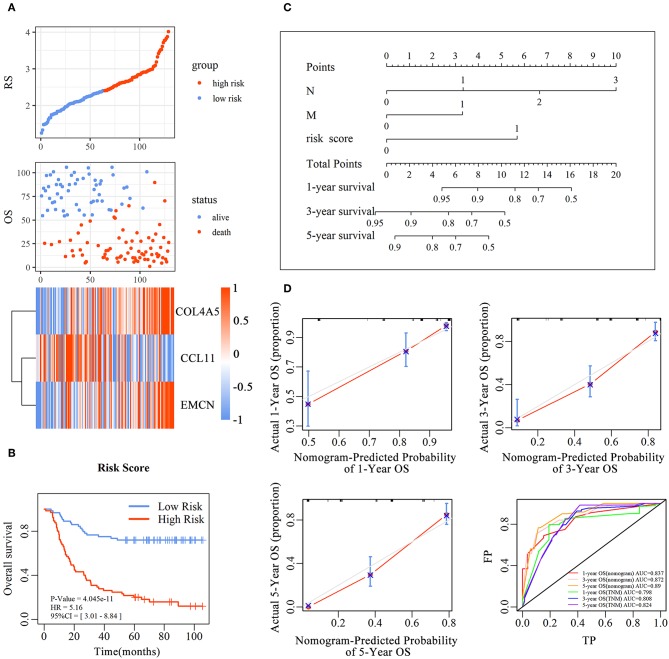
The predictive value of the risk score for diffuse type GC. The association between RS and OS, survival status and the expression of genes in the 3-gene signature was showed in scatter and heatmap plot **(A)**. The pseudocolors on the right of heatmap plot represent expression levels from low to high on a scale from −1 to 1, ranging from a low correlation power (white) to high (blue, or red). Kaplan–Meier was used to estimate the OS probability based on the RS in diffuse type GC, in which red plots indicates high-RS group, while the blue plot represents low-RS group **(B)**. Log-rank test was used to compare the survival distribution of these two groups. The nomogram was established with the RS, N-stage and M-stage in diffuse type GC **(C)**. The comparison between predicted and actual outcome for 1-, 3-, and 5-year survival probabilities in the nomogram was showed in the Calibration plots. Receiver operating characteristic (ROC) curves was used to compare the predictive ability of nomogram model and TNM stage for 1-, 3-, and 5-year survival probabilities **(D)**.

Based on the expressions of 3-gene signature of diffuse type GC and their corresponding coefficients, a risk score (RS) for diffuse type GC was calculated as follows:

$$\begin{array}{l} RS=\left[-0.2733\times \text{EXP}\left(\text{CCL}11\right)\right]+\left[0.1769\times \text{EXP}\left(\text{COL}4\text{A}5\right)\right]\\ \;+\left[0.4744\times \text{EXP}\left(\text{EMCN}\right)\right], \end{array}$$

and every patient was endowed with a RS. The distribution of RS for each sample, samples' survival status, and expression levels of genes in training set are illustrated in Figure 3A. With decrease of RS, the death events were accumulated and the expression levels of risk markers (coefficient > 0) were increased, while the protective markers were decreased. Furthermore, the Kaplan–Meier curves showed that patients in high-RS group presented a remarkably longer OS than that in low-RS group [hazard ratio (HR) = 5.01, 95% confidence interval (CI) = 3.01–8.84, *P* < 0.001] (Figure 3B).

To evaluate the prognostic value and identify the independent factors in diffuse type GC, univariate and multivariate COX regression analyses, involving RS and other clinicopathological characteristics, were performed. The results of univariate COX regression analysis showed that OS was significantly associated with the RS (HR = 5.16, 95%CI = 3.01–8.84, *P* < 0.001), T stage (HR = 1.73, 95%CI = 1.23–2.44, *P* < 0.001), N stage (HR = 2.62, 95%CI = 1.95–3.52, *P* < 0.001), and M stage (HR = 3.81, 95%CI = 2.20–6.61, *P* < 0.001). Meanwhile, the results of multivariate COX regression analysis indicated that RS (HR = 3.39, 95%CI = 2.25–5.09, *P* < 0.001), N stage (HR = 2.32, 95%CI = 1.72-3.16, *P* < 0.001), and M stage (HR = 2.346, 95%CI = 1.34–4.12, *P* < 0.01) were independent predictive factors (Table 2). Then, an independent factor nomogram model based on the independent predictive factors, including RS, N state, and M stage, was established for the prognostic prediction in patients with diffuse type GC. Figure 3C displayed that overall score could be measured to estimate the survival prognosis (1-, 3-, and 5-year survival probabilities), and the C-index of this nomogram model was 0.781 (95%CI = 0.732–0.83). The nomogram and actual observations in calibration curve showed a satisfactory overlap, indicating an optimal agreement (Figure 3D). Taken together, nomogram model based on RS appropriately predicted the prognosis of diffuse type GC.

**Table 2:** The univariate and multivariate Cox regression analysis between RS and other clinical characteristics and OS in diffuse type GC.

	**Variate COX**				**Multivariate COX**			
	**Coef**	**HR**	***P***		**Coef**	**HR**	***P***	
Sex	−0.2759	0.7589	0.2389					
Age	0.0037	1.0037	0.7016					
T	0.5508	1.7347	0.0016	**	0.0372	1.0379	0.8305	
N	0.9641	2.6224	<0.001	***	0.7609	2.1402	<0.001	***
M	1.3385	3.8133	<0.001	***	0.7513	2.1198	0.0094	**
RS	1.6409	5.1598	<0.001	***	1.3075	3.6969	<0.001	***

^***^P < 0.001; ^**^P < 0.01.

The analyses mentioned above were also carried out in intestinal type GC. It was uncovered that high-RS was related to long-time OS, which was inconsistent with that observed in diffuse type GC (Figures S1A,B); besides, RS, age, T stage, and N stage were found as independent predictive factors (Table S7). The nomogram model (Figure S1C) with the C-index of 0.786 (95%CI = 0.730–0.842) and a relatively accurate internal validation (Figure S1D) could predict the survival probabilities of patients with intestinal type GC.

All these results indicated that a subtype of multi-gene signature could accurately predict the prognosis of diffuse- and intestinal type GC, respectively.

### External Validation by Independence Analysis of Diffuse Type and Intestinal Type GC

**Figure 4:**
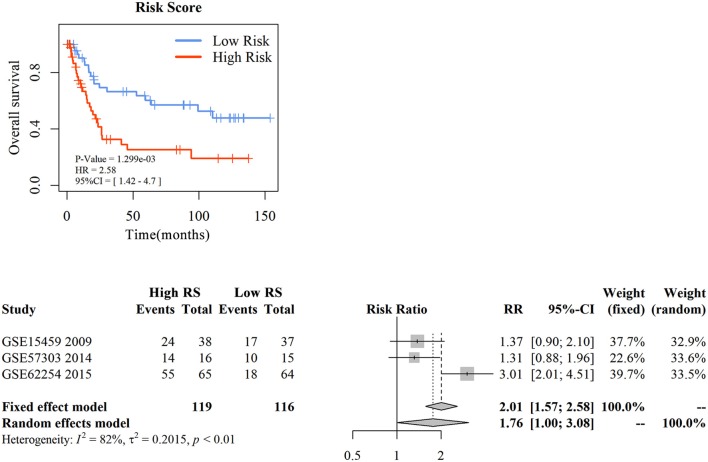
The external validation of nomogram in diffuse type gastric cancer. KM analysis of the RS for OS in diffuse type GC in GSE15459 was based on Log-rank test **(A)**. A GEO meta-analysis was used to valid the predictive of the RS in diffuse type GC **(B)**.

To assess the prognostic prediction value of 3-gene signature-derived RS in diffuse type GC and 5-gene signature-derived RS in intestinal type GC, GEO database was searched and Kaplan–Meier analysis was performed for the external validation. The result of diffuse type GC revealed that high-RS1 group presented significantly shorter OS than that in low-RS1 group (HR = 1.92, 95% CI = 1.02–3.59, *P* = 0.04), which was similar with the result obtained from GSE62254, illustrating a significant influence of the prognostic signature on the prognosis of patients with diffuse type GC (Figure 4A). However, for intestinal type, the OS in patients with high-RS was notably shorter than that with low-RS (HR = 2.91, 95% CI = 1.51–5.33, *P* < 0.001), which was inconsistent with the result of GSE62254 dataset (Figure S2).

Then, with a total searching of 15 GEO series containing Lauren subtype data, three series with integral survival data were screened, and a meta-analysis was conducted for further evaluation of 3-gene signature in prognostic prediction of diffuse type GC. As depicted in Figure 4B, the frequency of death events was increased in patients with high-RS compared with those with low-RS (OR = 5.42, 95% CI = 3.06–9.60, *P* < 0.01) although a slight heterogeneity was noted (I^2^ = 78%, *P* < 0.01). A random effects model showed that patients with high-RS might be associated with a high death risk in diffuse type GC (OR = 4.83, 95% CI = 1.19–15.90, *P* < 0.01). These data strongly suggested that 3-gene signature-derived RS could appropriately predict the prognosis of patients with diffuse type GC.

### Analysis of Correlation Between 3-Gene Signature and Clinical-Pathological Parameters in Diffuse Type GC

With analysis of correlation between 3-gene signature and clinical pathological parameters in diffuse type GC, we found that high-RS was markedly associated with the high N stage (*P* = 0.027), peritoneal-seeding metastasis (*P* < 0.001), and malignant ascites (*P* < 0.001) (Table 3). Then, the association of every single gene of the 3-gene signature and metastasis was also analyzed in diffuse type. As shown in Table 4, both EMCN and COL4A5 were positively correlated with peritoneal-seeding metastasis (*P* < 0.001) and malignant ascites (*P* = 0.001), whereas CCL11 could inhibit liver metastasis (*P* = 0.055). These results indicated that high-RS, especially high expression levels of EMCN and COL4A5, might be involved in peritoneal metastasis of diffuse type GC.

**Table 3:** The correlation between RS and clinical pathological parameters in diffuse type GC.

		**Low-RS**	**High-RS**	***P***	
Age	<60	36	28	0.1868	
	>60	28	37		
Gender	Female	27	31	0.6517	
	Male	37	34		
T	2	35	27	0.2944	
	3	24	33		
	4	5	5		
N	0	6	2	0.0265	*
	1	32	21		
	2	16	20		
	3	10	22		
M	0	59	52	0.0813	
	1	5	13		
Peritoneal seeding	No	56	30	<0.001	***
	Yes	8	35		
Ascites	No	58	33	<0.001	***
	Yes	6	32		
Liver	No	61	57	0.2171	
	YES	3	8		
Distant lymph node	No	64	63	0.4829	
	Yes	0	2		
Bone	No	63	61	0.3710	
	Yes	1	4		

^***^P < 0.001; ^*^P < 0.05.

**Table 4:** The correlation of EMCN, COL4A5, and CCL11 with the clinical pathological parameters related to metastasis in diffuse type GC.

		**Low-EMCN**	**High-EMCN**	***P***	
Peritoneal seeding	No	54	32	0.0001	***
	Yes	10	33	0.0001	
Ascites	No	54	37	0.0013	**
	Yes	10	28	0.0013	
Liver	No	57	61	0.5109	
	Yes	7	4	0.5109	
Distant lymph node	No	62	65	0.4692	
	Yes	2	0	0.4692	
Bone	No	61	63	0.9859	
	Yes	3	2	0.9859	
		**Low-COL4A5**	**High-COL4A5**	***P***	
Peritoneal seeding	No	51	35	0.0034	**
	Yes	13	30	0.0034	
Ascites	No	55	36	0.0003	***
	Yes	9	29	0.0003	
Liver	No	52	54	0.9673	
	Yes	12	11	0.9673	
Distant lymph node	No	61	57	0.2171	
	Yes	3	8	0.2171	
Bone	No	63	61	0.3710	
	Yes	1	4	0.3710	
		**Low-CCL11**	**High-CCL11**	***P***	
Peritoneal seeding	No	40	46	0.4183	
	Yes	24	19		
Ascites	No	44	47	0.8026	
	Yes	20	18		
Liver	No	55	63	0.0551	
	Yes	9	2		
Distant lymph node	No	62	65	0.4692	
	Yes	2	0		
Bone	No	61	63	0.9859	
	Yes	3	2		

^***^P < 0.001; ^**^P < 0.01.

### The Effect of 3-Gene Signature on Peritoneal Metastasis in Diffuse Type of GC

**Figure 5:**
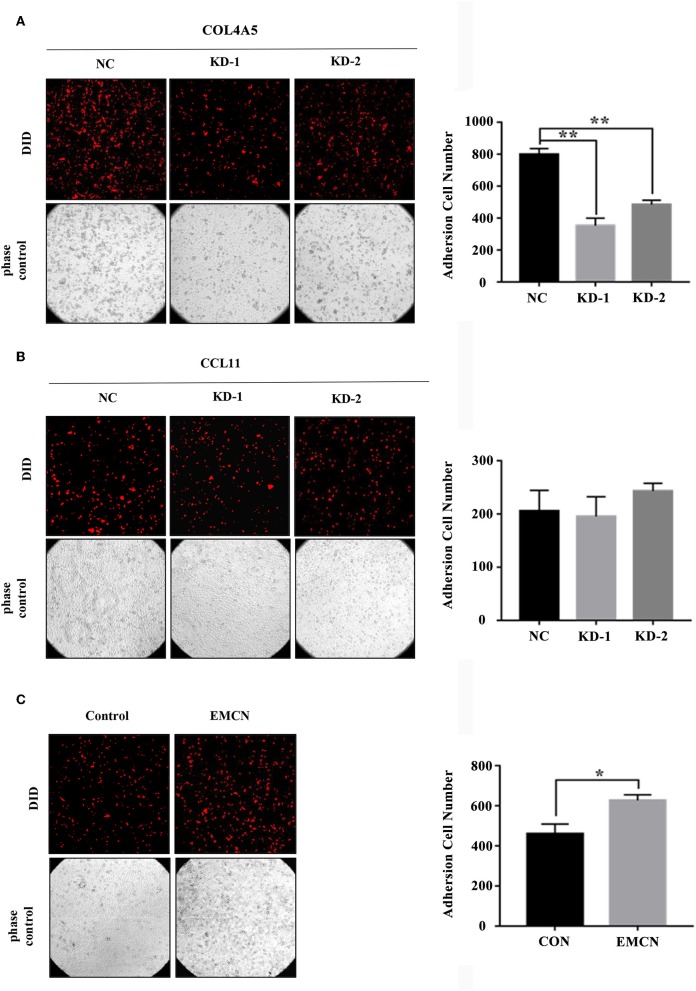
The effect of COL4A5, CCL11, and EMCN on the adhesion ability of diffuse type GC cells on peritoneal mesothelial cells. After transfected with siCOL4A5 **(A)** or siCCL11 **(B)**, or treated with EMCN **(C)**, the adhesion ability of MKN45 cells on HMR-SV5 cells was observed by Light microscopy. The columns on the right represent the cell numbers by counting three fields, and error bars represent the mean ± SD of three independent experiments. ^*^P < 0.05; ^**^P < 0.01.

It was revealed that adhesion of GC cells to mesothelium is an important step in peritoneal metastasis. Therefore, to investigate the role of EMCN, COL4A5, and CCL11 in peritoneal metastasis, diffuse type GC cell lines, MKN45 and NUGC4, were used to detect their adhesion abilities to HMV-SV5 cells. The findings showed that after transient transfection of siRNAs targeted to COL4A5 or CCL11 into MKN45 (Figure S3A) and NUGC4 (Figure S3B), the adhesion ability of COL4A5-KD cells was significantly decreased (Figure 5A and Figure S4A), whereas no change was observed in CCL11-KD cells (Figure 5B and Figure S4B). Then, 10 ng/ml of EMCN (ProSpec-Tany TechnoGene Ltd., Israel), which is a factor mainly secreted from endothelia, was used to pre-treat MKN45 and NUGC4 cells for 24 h, followed by detection of the adhesion ability of diffuse type GC cells to SV5 cells. As a result, EMCN significantly increased the adhesion of MKN45 and NUGC4 cells (Figure 5C and Figure S4C). These results indicated that among the 3 genes of the signature, COL4A5 and EMCN could promote peritoneal metastasis in diffuse type GC.

### COL4A5 Activated Wnt Signaling Pathway in Diffuse Type GC

**Figure 6:**
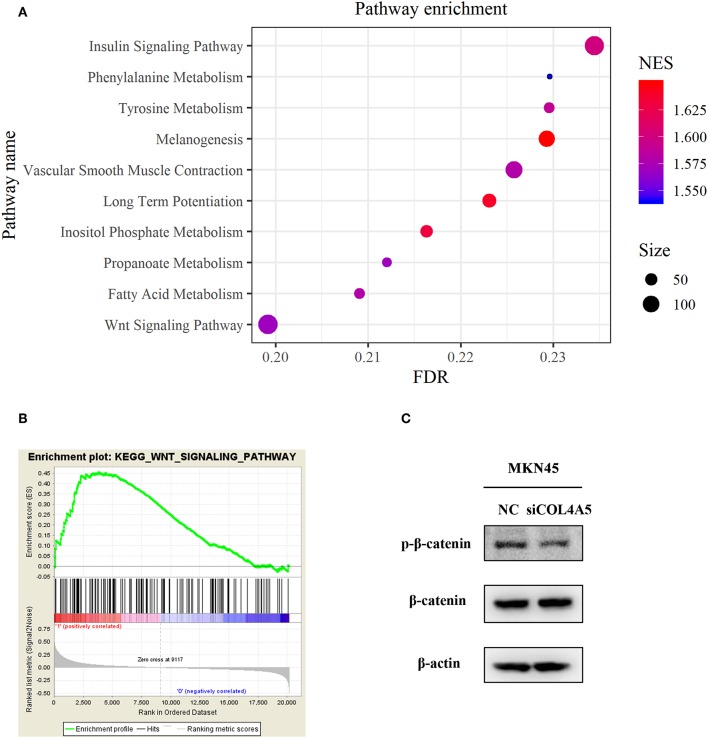
Functional enrichment analysis of COL4A5 in diffuse type GC. KEGG **(A)** and GSEA **(B)** analysis were used to analyze COL4A5 high expression group enriched signaling pathways. The expression of p-β-catenin and β-catenin was detected by western blot after transient knockdown of COL4A5. β-actin was used as internal control **(C)**.

For a further exploration of mechanism under COL4A5-promoted peritoneal metastasis in diffuse type GC, gene set enrichment analysis (GSEA) was conducted. The result showed that COL4A5 high expression group was remarkably enriched in “Melanogenesis,” “Long-term potentiation,” “Insulin signaling pathway,” “Vascular smooth muscle contraction,” “Tyrosine metabolism,” “Fatty acid metabolism,” “Propanoate metabolism,” “Wnt signaling pathway,” and “Phenylalanine metabolism,” indicating that COL4A5 might promote peritoneal metastasis via “Wnt signaling pathway” in diffuse type GC (Figures 6A,B). Knockdown of COL4A5 in MKN45 cells decreased the phosphorylation level of β-catenin, the key gene of “Wnt signaling pathway,” further suggesting that Wnt signaling pathway might be involved in COL4A5-promoted peritoneal metastasis (Figure 6C).

### GSEA of EMCN Was Related to Peritoneal Metastasis in Diffuse Type GC

**Figure 7:**
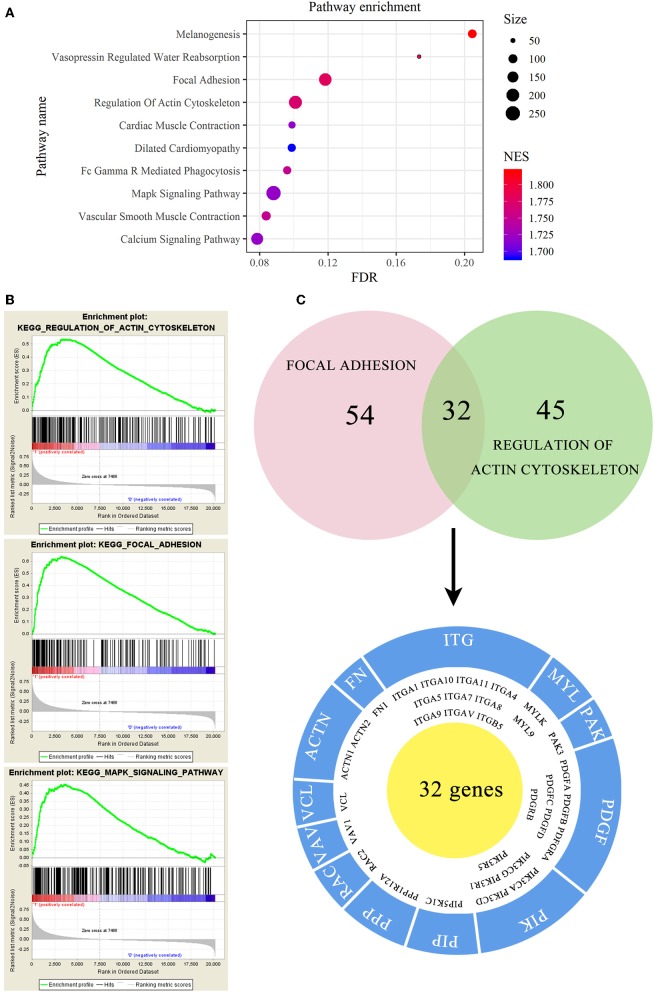
Functional enrichment analysis of EMCN in diffuse type GC. EMCN high expression group-enriched signaling pathways were analyzed using KEGG **(A)** and GSEA **(B)**. Venn diagram analysis showed coexpression genes in focal adhesion and regulation of actin cytoskeleton and mainly focused on integrin family **(C)**.

To investigate the mechanism of EMCN on the promotion of peritoneal metastasis in diffuse type GC, GSEA was used to analyze the possible functions of EMCN. As shown in Figure 7A, high expression of EMCN was mainly enriched in “Melanogenesis,” “Vasopressin regulated water reabsorption,” “Focal adhesion,” “Regulation of actin cytoskeleton,” “FC gamma R-mediated phagocytosis,” “Vascular smooth muscle contraction,” “Cardiac muscle contraction,” “MAPK signaling pathway,” “Calcium signaling pathway,” and “Dilated cardiomyopathy,” indicating that ECMN might promote peritoneal metastasis of the diffuse type GC by adhesion and invasion-related pathways, such as “Focal adhesion,” “Regulation of actin cytoskeleton,” and “MAPK signaling pathway” (Figure 7B). Subsequently, 32 genes both in “Focal adhesion” and “Regulation of actin cytoskeleton” pathway, which were known to be closely related to peritoneal metastasis, were further classified into 12 gene families. Among these 32 genes, integrin family, including 10 members, was the largest family, indicating integrin family might play an important role in EMCN-promoted peritoneal metastasis (Figure 7C).

### EMCN Activated Integrins-FAK Pathway in Diffuse Type GC

**Figure 8:**
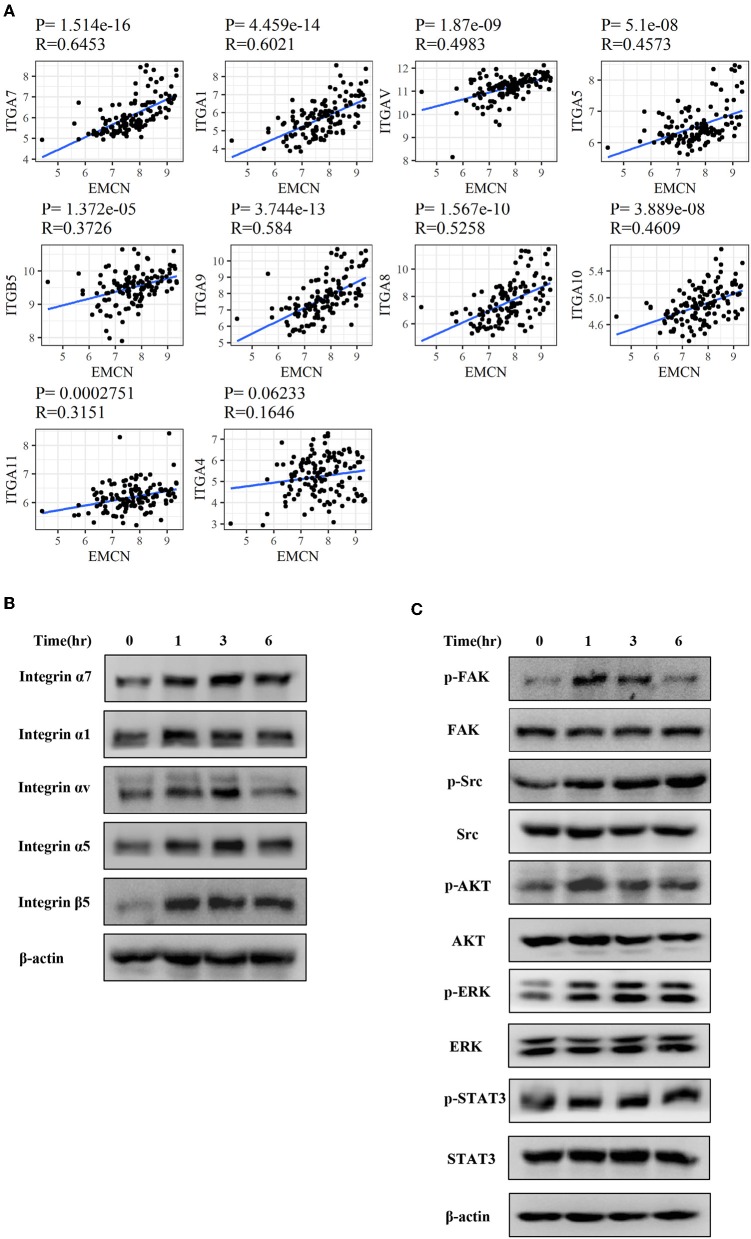
EMCN activated integrin-FAK pathway. The correlation between the expression of integrin family members and EMCN was analyzed using GSE62254 dataset **(A)**. Expression change of integrin family members, integrin α1, α5, α7, αv, and β5 induced by EMCN (100 ng/ml) was detected by western blot in MKN45 cells. β-actin was used as internal control **(B)**. After MKN45 cells were treated with EMCN, the expression of p-FAK, FAK, p-Src, Src, p-AKT, AKT, p-ERK ERK, p-STAT3, and STAT3 were detected by western blot. β-actin was used as internal control **(C)**.

To further investigate whether integrin family was involved in EMCN-promoted peritoneal metastasis, GSE62254 data were used to analyze the correlation between integrin family and EMCN expression. Ten members of integrin family, which overlapped in “Focal adhesion” and “Regulation of actin cytoskeleton” pathway, were all positively correlated with EMCN (Figure 8A). Then, the effect of EMCN on the expression change of several integrin members and their downstream pathways were detected by western blot. The result showed that 100 ng/ml EMCN significantly upregulated the expression of integrin α1, α5, α7, αv, and β5 in MKN45 cells (Figure 8B), as well as dramatically increased the phosphorylation levels of FAK, Src, AKT, ERK, and STAT3, the potential downstream pathway of integrin (Figure 8C). All these results suggested that EMCN might promote peritoneal metastasis through activating integrin-FAK pathway.

## Discussion

In the present study, 3-gene signature was identified for diffuse type GC, and high-RS based on 3-gene signature exhibited a significantly increased risk of short OS. Furthermore, a nomogram model based on 3-gene signature for prognostic prediction of diffuse type was established, and uncovered that EMCN and COL4A5 were highly involved in peritoneal metastasis of diffuse type GC.

In the current study, the LASSO algorithm combined with the AIC was used to select an optimal prognostic signature with the smallest number of gene markers for identification of the 3-gene signature in diffuse type GC. LASSO generally minimizes residual sum of squares (RSS) to a constraint on the absolute size of coefficient estimates. AIC is an information-based criterion to select a model based on the minimum distance between the logarithms of the likelihood and Kullback–Leibler information. COX regression model with AIC can be applied when the number of independent variables is notably less than the number of samples (<1/10). However, as there are several independent variables, even the number to be more than the number of samples, the LASSO needs to a complementary algorithm to shrink the dimension induced by exceeded independent variables. The combination of LASSO and AIC not only could increase the precision and efficiency of variable selection and reduce the dimension of prognostic models, but also could avoid the over-fitting in prediction and estimation. Therefore, the combination of LASSO and AIC was herein applied in diffuse type GC, whereas calculation of AIC alone was used in intestinal type GC.

At present, in addition to the TNM stage, multi-gene prognosis signatures, including mRNAs or non-coding RNAs, were also developed to assess the prognosis of GC patients. However, to date, no study has concentrated on Lauren subtype-specific multi-gene signature to evaluate prognosis of GC. Therefore, in the present study, we explored the 5-gene signature in intestinal type GC and 3-gene signature in diffuse type GC, which were fully different from each other and could evaluate subtype-specific prognosis of GC patients. Furthermore, the prognostic predictive model of 3-gene signature was proved to be able to accurately investigate the prognosis of diffuse type GC. This model might be applied to identify the high-risk patients, and assess the prognosis, so as to facilitate the precise treatment in diffuse type GC.

The 3-gene signature identified in diffuse type GC included COL4A5, EMCN, and CCL11. COL4A5 is one of the major components of the glomerular basement membrane, and its mutation or aberration is involved in Alport syndrome and uterine leiomyomas (23). It was also revealed that COL4A5 was down-regulated in colorectal cancer due to the hypermethylation of its promoter region (24). EMCN, which is specifically expressed in endothelial cells on the surface of the capillary and venous, is known to be involved in vascular endothelial growth factor (VEGF)-induced angiogenesis via VEGF receptor 2 (VEGFR2) (25). The role of EMCN in cancer has still remained controversial, as EMCN is highly expressed in lung cancer (26), whereas is downregulated in a primary central nervous system lymphoma (27). The chemokine CCL11, acting as selective eosinophil chemo-attractant, was found to be derived from fibroblast and tumor cells (28), and could be highly expressed in ovarian cancer and prostate cancer (29). However, the role of these genes in GC still remains obscure. In the current study, we found that COL4A5 and EMCN, rather than CCL11, could promote peritoneal metastasis by enhancing the adhesion ability of diffuse type GC cells to mesothelial cells. Moreover, further study revealed the molecular mechanisms of these genes in peritoneal metastasis of diffuse type GC. COL4A5-activated Wnt signaling pathway, and EMCN-activated FAK-AKT/ERK/STAT3 signaling pathway through upregulating integrin family might be involved in peritoneal metastasis of diffuse type GC. Therefore, this study indicated that not only diffuse type GC, but also the tumor microenvironment is involved in the promotion of peritoneal metastasis, which may justify poor OS of diffuse type GC. However, further study needs to be conducted to investigate the molecular mechanisms of these genes in peritoneal metastasis of diffuse type GC.

The present study contains several limitations. Firstly, this study was conducted based on genomics rather than proteomics, which might affect the accuracy of signature prediction to a certain extent. Therefore, it is necessary to detect the expression of three genes in another larger sample size of diffuse type GC patients to validate the predictive abilities of the 3-gene signature for diffuse type GC in the future. Secondly, it was difficult to popularize the application of multi-genome sequencing in clinical practice due to its price and practicality. With the development of liquid biopsy technology, the clinical predictive value of 3-gene signature maybe further easily applied in future research. Thirdly, the identification of 5-gene signature in intestinal type GC could not be validated in other datasets. Therefore, a robust detection method needs to be developed for the prognostic prediction in diffuse type GC; meanwhile, multi-gene signature of intestinal type GC needs to be explored in further studies with a larger sample size.

In summary, the current research not only revealed the molecular difference between intestinal- and diffuse type GC, but also demonstrated that 3-gene signature could effectively predict the survival of patients with diffuse type GC. The identification of prognosis signature in diffuse type might provide a novel therapeutic approach to evaluate the prognosis of GC patients based on Lauren classification system and the expression level of 3-gene signature. Additionally, the RS, EMCN, and COL4A5, could promote the peritoneal metastasis process of GC cells partially through Wnt and integrin-FAK signaling pathway at least. This study proposed a new approach for the application of bioinformatics in GC.

## Data Availability Statement

Publicly available datasets were analyzed in this study. This data can be found here: GSE62254, GSE15459, GSE57303.

## Author Contributions

BB and CZ analyzed the data and drafted the manuscript. BY, YJ, and KH helped interpreted the data. ZL and XZhe prepared all figures. XZha and SY completed adhesion assay. YF and XQ edited all tables. XC and YL designed the study and revised the manuscript. All authors read and approved the final manuscript.

### Conflict of Interest

The authors declare that the research was conducted in the absence of any commercial or financial relationships that could be construed as a potential conflict of interest.

## Abbreviations

GC: gastric cancer
GEO: Gene Expression Omnibus
DEGs: differentially expressed genes
FC: fold change
RS: risk score
LASSO: Least Absolute Shrinkage and Selection Operator
AIC: the Akaike information criterion
HR: hazard ratio
CI: confidence interval
TNM: Tumor Node Metastasis
OS: overall survival.
