# Supplementary material for: Identification of Subtype-Specific Three-Gene Signature for Prognostic Prediction in Diffuse Type Gastric Cancer
Source: Front Oncol. 2019 Nov 12;9:1243. doi: 10.3389/fonc.2019.01243 (PMC6869510; doi:10.3389/fonc.2019.01243)
Supplement: Supplementary file 8 [file Data_Sheet_1.PDF]

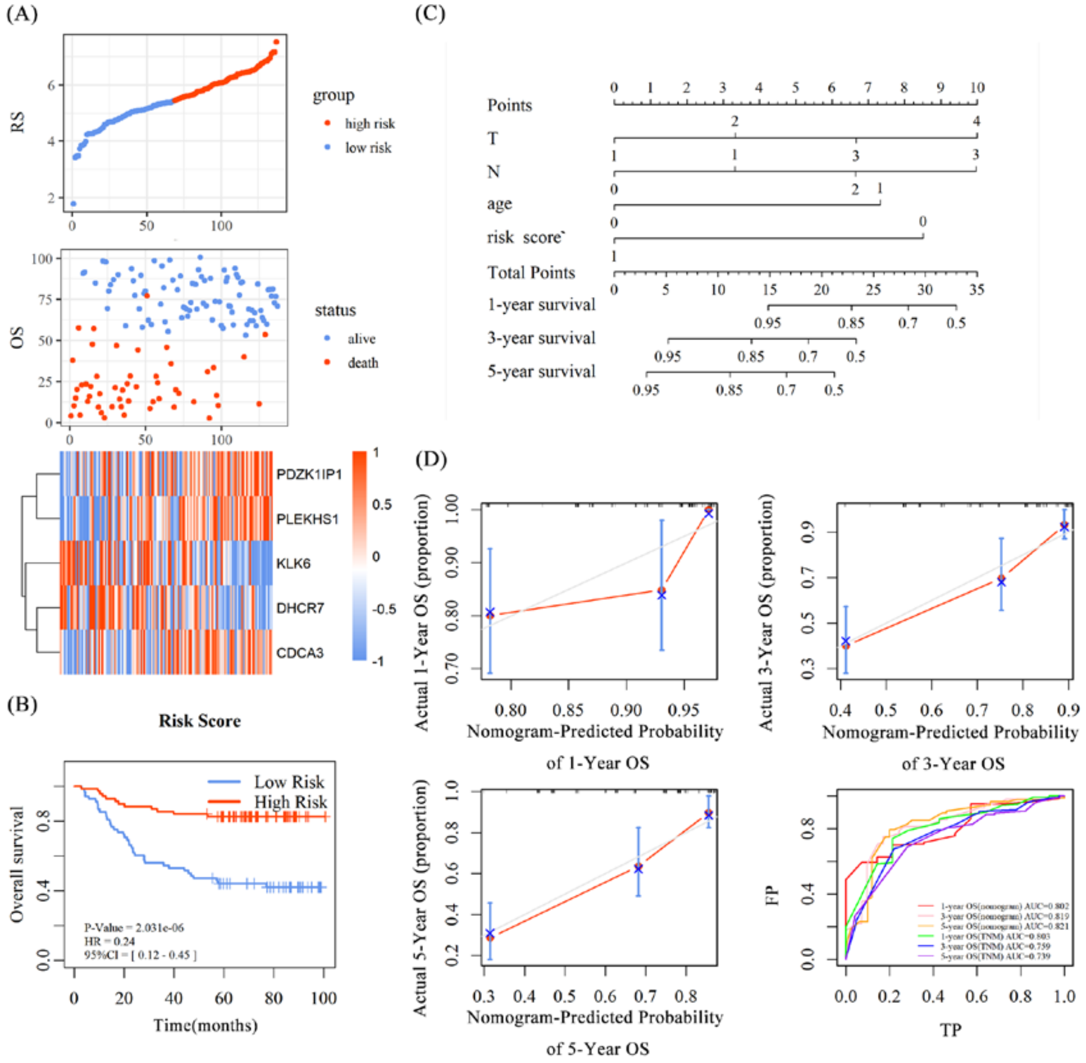

Supplementary Figure 1. The association between RS and OS, survival stauts and the expression of genes in the 5-gene signature was showed in scatter and heatmap plot (A). Kaplan–Meier was used to estimate the OS probability based on the RS in intestinal type GC. (B). The nomogram was established with the RS, age, T-stage and N-stage in diffuse type GC (C). The comparison between predicted and actual outcome for 1-, 3- and 5-year survival probabilities in the nomogram was showed in the Calibration plots.

Receiver operating characteristic (ROC) curves was used to campare the predictive ability of nomogram model and TNM stage for 1-, 3- and 5-year survival probabilities (D).
