# Supplementary material for: Identification of Subtype-Specific Three-Gene Signature for Prognostic Prediction in Diffuse Type Gastric Cancer
Source: Front Oncol. 2019 Nov 12;9:1243. doi: 10.3389/fonc.2019.01243 (PMC6869510; doi:10.3389/fonc.2019.01243)
Supplement: Supplementary file 9 [file Data_Sheet_2.PDF]

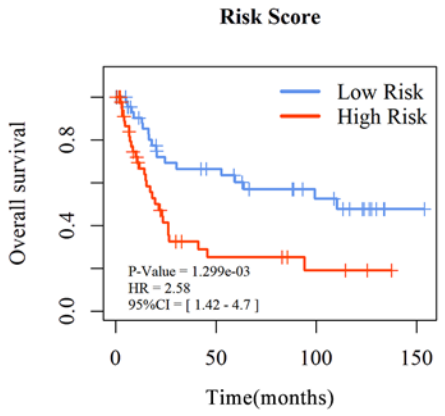

Supplementary Figure 2. KM analysis of the RS for OS in intestinal type GC in GSE15459 was based on Log-rank test.
