# Supplementary material for: Identification of Subtype-Specific Three-Gene Signature for Prognostic Prediction in Diffuse Type Gastric Cancer
Source: Front Oncol. 2019 Nov 12;9:1243. doi: 10.3389/fonc.2019.01243 (PMC6869510; doi:10.3389/fonc.2019.01243)
Supplement: Supplementary file 10 [file Data_Sheet_3.PDF]

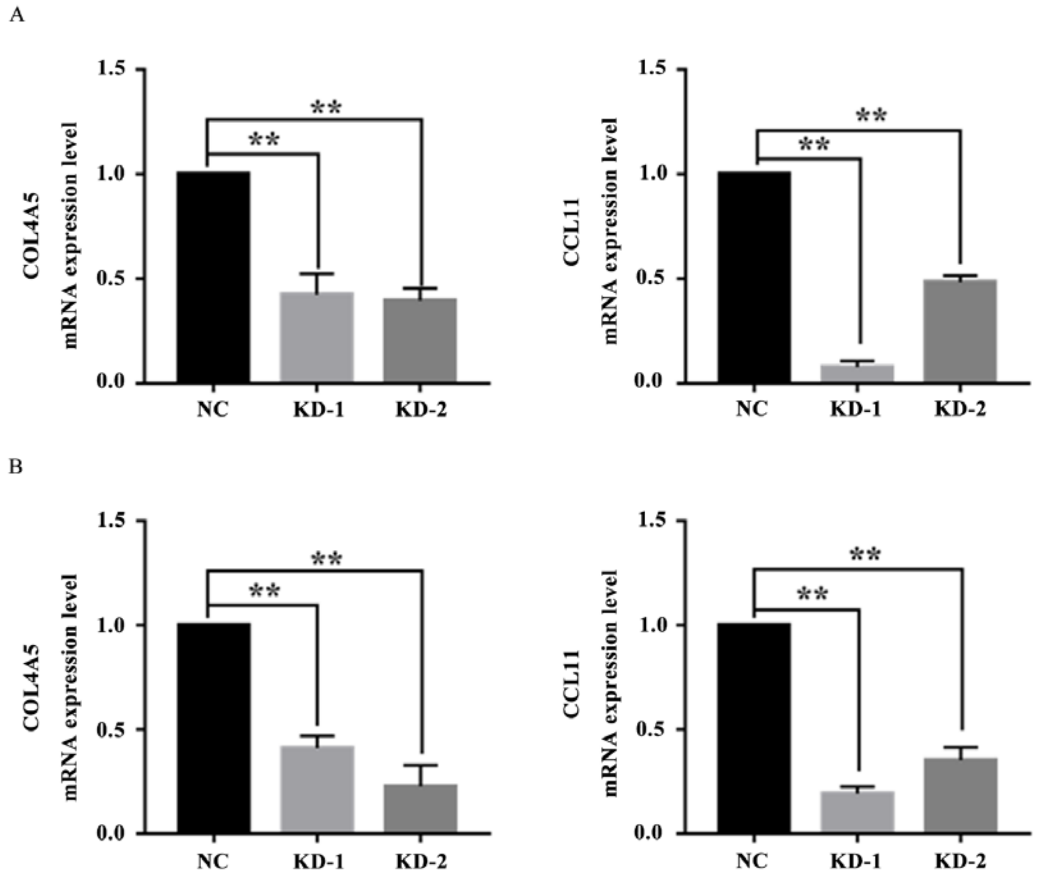

Supplementary Figure 3. Transient transfection efficiency of COL4A5 and CCL11 in MKN45 (A) and NUGC4 (B) cells was detected by qRT-PCR.
