# Supplementary material for: Identification of Subtype-Specific Three-Gene Signature for Prognostic Prediction in Diffuse Type Gastric Cancer
Source: Front Oncol. 2019 Nov 12;9:1243. doi: 10.3389/fonc.2019.01243 (PMC6869510; doi:10.3389/fonc.2019.01243)
Supplement: Supplementary file 11 [file Data_Sheet_4.pdf]

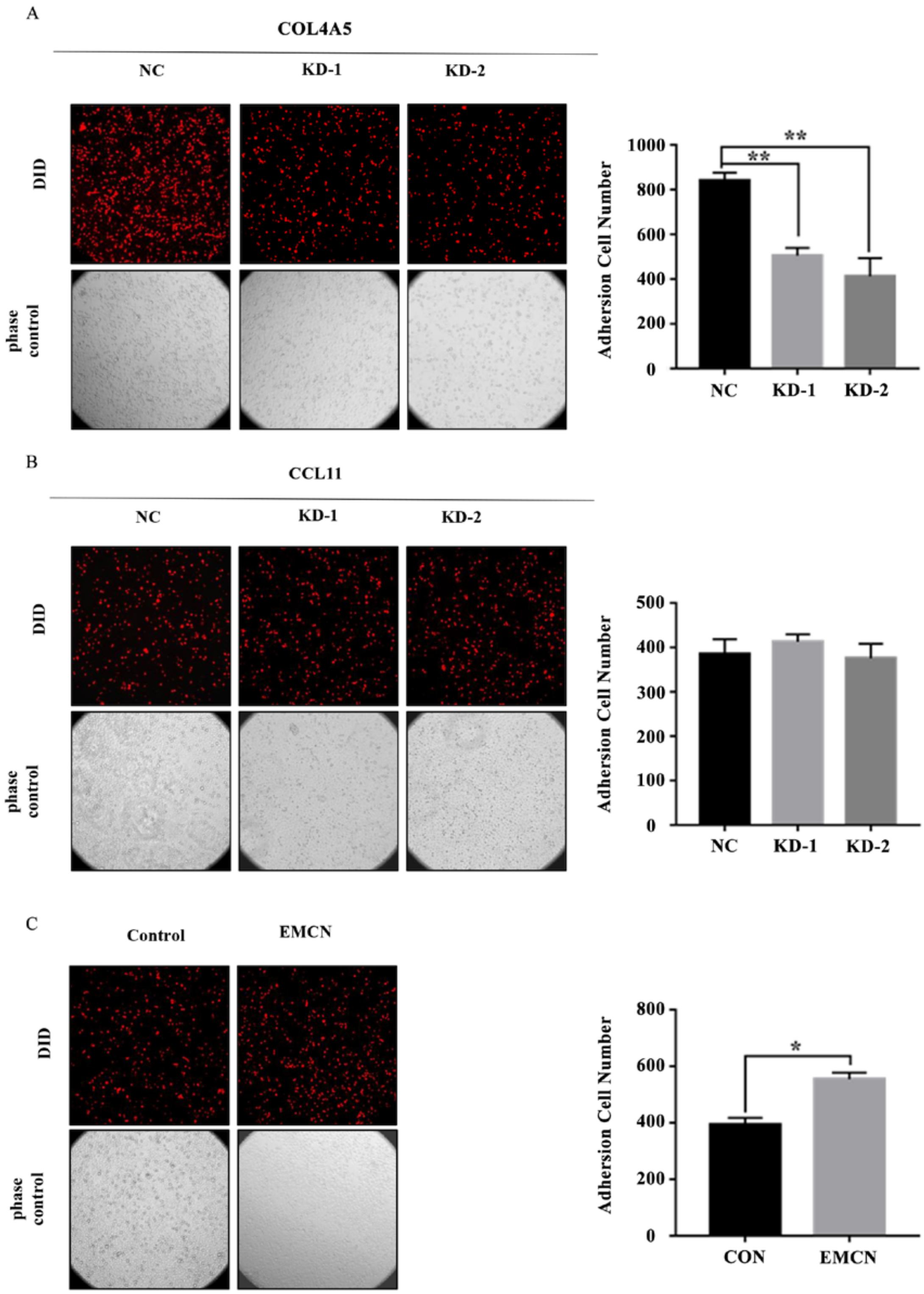

Supplementary Figure 4. The effect of COL4A5, CCL11 and EMCN on the adhesion ability of diffuse type GC cell line NUGC4 on peritoneal mesothelial cells.
